# Contributions of Retinoid Signaling to Autism-like Behaviors Induced by Early Postnatal Lead Exposure in the Mouse Cerebellum

**DOI:** 10.3390/cimb47100861

**Published:** 2025-10-18

**Authors:** Xiaochun Xia, Xulan Zhou, Zihan Ma, Li Liu, Yaqi Wang, Yongli Wu, Ying Zhang, Juan Wang

**Affiliations:** 1 Department of Public Health and Medical Technology, Xiamen Medical College, Xiamen 361023, China; xiaxiaochun@xmmc.edu.cn (X.X.); zxl@xmmc.edu.cn (X.Z.); zihan_ma0317@163.com (Z.M.); wangyaqi@xmmc.edu.cn (Y.W.); wyongli1031@163.com (Y.W.); hizy6155@163.com (Y.Z.); 2 School of Public Health, Fujian Medical University, Fuzhou 350122, China; 990712@fjmu.edu.cn

**Keywords:** autism spectrum disorder (ASD), lead (Pb), cerebellum, retinoid signaling, postnatal

## Abstract

Autism spectrum disorder (ASD) is a group of neurodevelopmental dysfunctions characterized by a heterogeneous etiology that involves gene–environment interactions. Early postnatal lead (Pb) exposure has been found to be associated with the etiology of ASD, but the mechanisms remain unclear. The present study aims to investigate the effects of early Pb exposure on the emergence of ASD-like behaviors in offspring and to evaluate its potential relationship with morphological changes and underlying mechanisms in the cerebellum. The study established a mouse model to study early postnatal Pb exposure and examined ASD-like behaviors through the open field test, novel object recognition test, marble burying test, and three-chamber social test. Quantification of Pb levels was performed in cerebellar tissue, examination of Purkinje cell morphology was carried out, and identification of differential protein expression was conducted using TMT-based quantitative proteomics. The study revealed that the offspring of Pb-exposed mice showed significant social deficits, increased repetitive behaviors, and cognitive impairments. The cerebellum showed both elevated Pb levels and a reduction in Purkinje cells. Proteomic analysis identified 45 proteins that were differentially expressed, showing disruption in the retinoid signaling pathway. These findings demonstrate that early postnatal Pb exposure leads to ASD traits and that retinoid signaling may be a key pathway in the cerebellum, at least in part.

## 1. Introduction

Lead (Pb) is a heavy metal with well-established neurotoxicity and poses a significant global health risk, particularly to children [1]. Children exposed to Pb during critical developmental stages exhibit a higher prevalence of neurodevelopmental disorders, such as autism spectrum disorder (ASD) [2,3,4], and are more likely to have lower IQ scores [5,6]. Consistent with previous studies [7,8], our previous study demonstrated a positive correlation between Pb levels and the severity of symptoms in children with ASD [9]. Animal experiments also showed that Pb exposure exacerbates immunological dysfunctions associated with ASD, which indicated that Pb exposure may increase the risk of ASD [10,11]. Therefore, it is very necessary to strengthen research into the mechanisms linking early-life Pb exposure to the onset and progression of ASD, which is crucial for developing preventive strategies against neurodevelopmental disorders and improving children’s health worldwide.

Pb can reach and accumulate in various tissues and organs, including the brain, liver, kidneys, lungs, spleen, muscles and heart [11,12,13,14,15]. The brain is particularly vulnerable to exogenous toxicants during critical developmental windows, especially in the early postnatal period [16,17]. Notably, Pb exhibits high toxicity to the developing brain due to its ability to cross the blood–brain barrier (BBB). Therefore, Pb toxicity is implicated in various neurological disorders, with sub-chronic exposure being a potential contributor to cerebral dysfunction [18]. Research showed that Pb exposure can cause specific damage to different regions of the brain, including the hippocampus, frontal lobe cortex, cerebellum, and striatum [19]. Moreover, Pb can induce non-spatial memory deficits and impair synaptic plasticity in hippocampal CA1 excitatory neurons [20]. Specifically, Pb-induced neurotoxicity in the cerebellum manifests through multiple mechanisms, including structural injury, oxidative stress, disrupted neurotransmission, and aberrant apoptosis [21]. Studies on ASD have mostly focused on abnormal hippocampal function. The cerebellum, traditionally associated with voluntary motor coordination, is increasingly recognized for its involvement in non-motor functions, including social behavior and cognitive processing [22,23]. The 36-fold elevated risk of ASD after perinatal cerebellar injury contributed to the confluence of these pathophysiological processes [24]. The developmental timeline of the cerebellum is similar in humans and rodents, and both species share an extended window of vulnerability around birth. The early post-natal period is highly susceptible to injury due to the multiple changes, including neurogenesis, neuronal migration and circuit formation, required for the proper maturation of the cerebellum [25]. Thus, the cerebellum may play a potential role in mediating the interaction between genetic susceptibility to ASD and environmental Pb exposure. Elucidating the role of cerebellar dysfunction in Pb-induced ASD could provide critical insights into the environmental etiology of neurodevelopmental disorders and reveal novel therapeutic targets. The underlying molecular mechanisms, especially those linking Pb-mediated cerebellar damage to ASD-like phenotypes, remain poorly understood.

Retinoids comprise both natural and synthetic compounds [26], and they play essential roles in physiological functions such as embryonic development [27,28]. The core of retinoids signaling pathway is retinoic acid (RA), which is locally synthesized in the cerebellum and plays an essential for neuronal development [29]. Regarding the pathway from retinol to all-trans RA in ASD, one study demonstrated that children with ASD exhibited altered regulation characterized by reduced serum atRA levels [30]. Specifically, serum RA levels were found to be decreased in the ASD group and showed a significant negative correlation with ASD severity [31]. Studies showed that RA replenishment could exert beneficial effects on autistic-like behaviors in fragile X syndrome (FXS), a condition characterized by impairments in social behavior [32]. Specifically, RA supplementation has been shown to rescue deficits in social novelty recognition. In VPA-induced ASD model rats, RA administration was found to ameliorate social deficits and modulated functional connectivity, especially in the hypothalamus and facial nerve regions [33]. Notably, it has been reported that Pb exposure can induce damage to the hypothalamus and pituitary of mice through activating the RA signaling pathway [34]. However, it remains unclear whether retinoid signaling contributes to the development of ASD-like behaviors in mouse model of Pb exposure.

Building upon these findings, the present study aimed to investigate the effects of early Pb exposure on the emergence of ASD-like behaviors, cerebellar morphological changes, and protein expressed alterations in offspring. We observed that Pb-exposed offspring exhibited significant ASD-like traits and abnormal Purkinje cell morphology in the cerebellum. Proteomic and bioinformatic analyses further revealed that disruption of the retinoic acid signaling pathway may represent a key mechanism underlying the etiology of Pb-induced ASD.

## 2. Materials and Methods

### 2.1. Establishment of Animal Models and Grouping

C57BL/6 mice were purchased from the Experimental Animal Centre of Xiamen University (Xiamen, China). The mice were placed in a room that had a fully controlled environment, with constant temperature and humidity, and a 12 h light/dark cycle. They were allowed to freely consume food and water. All animal experiments are in line with the Animal Research: Reporting of In Vivo Experiments (ARRIVE) guidelines and are carried out following the National Research Council’s Guide for the Care and Use of Laboratory Animals. The experiments followed the National Institutes of Health Guide for the Care and Use of Laboratory Animals and received approval from the Xiamen Medical College Animal Ethics Committee (Approval No. 2021041311, Approval date: 13 April 2021).

Male and female mice were mated overnight, and the vaginal secretion was collected the next morning. Pregnant females were brought up individually. The offspring’s birth day was specified as postnatal day 0 (PND0). The male offspring were randomly assigned to a control group, a low-Pb group (15 mg/kg bodyweight PbAc), and a high-Pb group (30 mg/kg bodyweight PbAc). The Pb exposure protocol was based on a previous study [35,36] and was designed to reflect environmentally relevant exposure levels documented in children with Pb poisoning. Male offspring were intraperitoneally injected with PbAc every two days from PND7 until PND21 to evaluate the effect of Pb during this critical period. The control group was given sterile 0.9% NaCl saline solution. Following Pb exposure, the male offspring were housed normally until they were used for further experiments.

### 2.2. ASD-like Behavior Tests

We conducted a series of tests to assess autism-like behaviors from PND35 to PND42, which included the three-chamber social test, marble burying test, novel object recognition test, and open field test. Each experimental group consisted of 8 male offspring.

#### 2.2.1. Open Field Test (OFT)

Based on established research protocols [37], the open field test was conducted on PND35 to evaluate psychomotor performance and anxiety-like behaviors in the experimental mice. Male offspring were placed in the central zone of the OFT arena (40 cm × 40 cm × 40 cm, length × width × height) and allowed to explore freely for 15 min. Their behavior was recorded via a camera mounted above the arena. The total distance traveled and time spent in the central zone were analyzed for each offspring using SMART software (Version 3.0.06). To prevent olfactory bias, the arena was thoroughly cleaned with 75% ethanol between trials.

#### 2.2.2. Novel Object Recognition (NOR) Test

The novel object recognition test was performed from PND36 to PND37, following a previously described method with minor modification [38]. An open field apparatus (40 cm × 40 cm × 40 cm) was used for the novel object recognition test. In the first session of the test, mice were presented with two similar objects (cylinders) and allowed to explore the open field freely for 5 min as a habituation session. Following a 24 h delay, in the second session, one of the two objects was replaced by a new object (cube) and then the test mouse was allowed to explore the open field freely for 5 min as an exploration session. The amount of time taken to explore the new object provides indexes of recognition memory, including discrimination index and preference index. Discrimination Index can be determined by the formula: [(Time Spent Exploring Novel Object − Time Spent Exploring Familiar Object)/(Time Spent Exploring Novel Object + Time Spent Exploring Familiar Object)]. To calculate the Preference Index, use the formula: [Time Spent Exploring Novel Object/(Time Spent Exploring Novel Object + Time Spent Exploring Familiar Object)] × 100%.

#### 2.2.3. Marble Burying Test

As detailed in a previous study [39], the marble burying test was employed on PND38 to evaluate repetitive behaviors in offspring mice. Each mouse was placed in a plastic chamber (40 cm × 40 cm × 40 cm) containing sterile woodchip bedding to a depth of 5 cm. Twenty glass marbles, previously sterilized with 90% alcohol, were arranged in an equidistant distribution across the testing area. Following an acclimation period, the mice were allowed to freely explore and bury marbles for 30 min. Upon completion of the test, the number of marbles buried was quantified. After each experiment, all marbles were thoroughly sterilized, and the testing chamber was replenished with fresh bedding.

#### 2.2.4. Three-Chamber Social Test

The three-chamber social test was performed from PND40 to PND41 to evaluate ASD-like social behaviors, in accordance with the methods described in our previous study [39]. The apparatus comprised a box containing three chambers (65 cm × 45 cm × 20 cm), each equipped with small rectangular openings to allow access between compartments. A wire mesh cage was placed in the middle chamber. During stage 1 of the experiment, an empty wire cage was placed in each of the two side chambers for adaptation. In the left chamber, an unfamiliar mouse was confined under the wire cage during the social preference test period. The right chamber contained an empty wire cage. In stage 3, another unfamiliar mouse was introduced under the wire cage in the right chamber for the social novelty preference test period. The test mouse was placed in the middle chamber during each stage and given 10 min to freely explore the apparatus. The duration of exploration directed toward the empty cage or the cage containing another mouse was recorded. Based on these measurements, the social index (SI) and social novelty preference index (SNI) were calculated. Data acquisition and analysis were performed using SMART software (Version 3.0.06).

### 2.3. Pb Level Determination in Cerebellum

Pb quantification in the cerebellum was performed as previously described with minor modifications [40,41]. At the end of autism-like behavior tests, cerebellar tissue was immediately collected (n = 3 per group). Samples (0.1–0.2 g) were digested in 5 mL of 65% nitric acid at room temperature for 6 h, followed by microwave-assisted digestion (MARSXpress, CEM Corporation, Matthewss, NC, USA). Pb levels were then quantified using inductively coupled plasma mass spectrometry (ICP-MS; Agilent 7500cx, Palo Alto, CA, USA).

### 2.4. Histopathological Analysis

Following behavioral testing, male offspring (n = 3 per group) were euthanized to assess cerebellar histopathology. Briefly, following deep anesthesia with ether, intracardiac perfusion was first performed with phosphate-buffered saline (PBS) until the effluent ran clear and the blood was completely replaced. The perfusate was then switched to 4% paraformaldehyde for fixation. Cerebellum was dissected, immediately fixed in 4% paraformaldehyde (overnight, 4 °C, 48 h), dehydrated in a gradient of ethanol, cleared in xylene, and embedded in paraffin (Leica AG, Frankfurt, Hessen, Germany). The tissues were cut into 5 μm sections, deparaffinized, and then stained with hematoxylin and eosin staining kit (H&E, Solarbio, G1120, Beijing, China), followed by mounting with neutral gum. Morphological changes in the cerebellum were examined in detail under light microscope (Olympus Co., Tokyo, Japan).

Quantitative analysis of Purkinje cells was conducted using Image J software (Version 1.54p). Purkinje cells were tallied by hand in each visual field. Each group was observed with two visual fields for every section, resulting in a total of six visual fields observed. Ultimately, the Purkinje cell counts in each group were subjected to statistical analysis.

### 2.5. Quantitative Proteomics Analysis

#### 2.5.1. Protein Extraction and Trypsin Digestion

Cerebellar protein extraction was performed according to established protocols with minor modifications [39,41]. Tissue samples were homogenized in lysis buffer using an automated homogenizer (JXFSTPRP-CL-BSC, Jingxin, Shanghai, China). The homogenate was centrifuged at 25,000× *g* for 15 min at 4 °C, and the precipitate was collected. Protein samples (100 μg) were subjected to reduction and alkylation, followed by precipitation with ice-cold acetone (5 × volume) at −20 °C for 2 h. Precipitated proteins were resolubilized with SDS-free lysis buffer and centrifugated at 25,000× *g* for 15 min at 4 °C. Protein concentration was determined by Bradford assay, and protein integrality was verified by SDS-PAGE.

For tryptic digestion, protein pellets were reconstituted in 100 μL of 50 mM TEAB buffer and digested with sequencing-grade modified trypsin (1:20 *w/w* enzyme-to-protein ratio) for 4 h at 37 °C. The reaction was terminated by acidification with 5 μL of 10% formic acid. Resulting peptides were lyophilized and stored at −80 °C until analysis.

#### 2.5.2. TMT Labeling

TMT labeling was conducted according to the manufacturer’s protocol (TMTpro™ 16plex Kit, Thermo Fisher Scientific, Waltham, MA, USA). Peptides (100 μg) were dissolved in 100 mM TEAB buffer. TMT reagents were equilibrated to room temperature and dissolved in 41 μL anhydrous acetonitrile (0.8 mg/vial). Dissolved TMT reagent was added to the peptide solution, followed by vortex mixing and incubation at room temperature for 2 h. The reaction was quenched with 5% hydroxylamine (8 μL, 15 min incubation). Labeled peptides were desalted using C18 solid-phase extraction. For fractionation, TMT-labeled peptides underwent separation by liquid chromatography (LC-20AB, Shimadzu, Tokyo, Japan) in conjunction with a Gemini C18 column (250 mm × 4.6 mm × 5 μm, Phenomenex, Torrance, CA, USA). Mobile phase A had 5% acetonitrile with a pH of 9.8, while mobile phase B had 95% acetonitrile at a pH of 9.8. Samples were reconstituted in mobile phase A and then separated at a flow rate of 500 µL/min using the following gradients: starting at 5% B for 10 min, increasing to 35% B over 40 min, further increasing to 95% B in 1 min, holding at 95% B for 3 min, and finally returning to 5% B for 10 min. The elution process was observed with a UV detector at 214 nm wavelength. Fractions were gathered every minute and combined into 20 fractions according to chromatographic peak profiles. The combined fractions were freeze-dried and kept at −80 °C.

#### 2.5.3. LC-MS/MS Analysis

Rehydration of the lyophilized polypeptides was carried out in mobile phase A (2% acetonitrile, 0.1% formic acid) followed by centrifugation at 20,000× *g* for 10 min. The supernatant (1 μL) was placed into a Thermo UltiMate 3000 UPLC system (Thermo Scientific, Germering, BY, Germany). Chromatographic separation was performed at a flow rate of 300 nL/min utilizing the subsequent gradient program: initiating at 5% mobile phase B (98% acetonitrile, 0.1% formic acid) for 5 min, then progressing to 25% B over 40 min, further increasing to 35% B over 5 min, a rapid increase to 80% B over 2 min, maintaining at 80% B for 2 min, and ultimately re-equilibrating at 5% B for 6 min. Peptides that were eluted from the LC column underwent ionization using a nanoESI source and were then analyzed on a Q-Exactive HF X tandem mass spectrometer (Thermo Fisher Scientific, San Jose, CA, USA) in data-dependent acquisition (DDA) mode. The main parameters were set as follows: ion source voltage at 2 kV, MS1 scanning range from 350 to 1500 *m/z*, resolution of 60,000, MS2 starting *m/z* at 100 with a resolution of 15,000. MS2 fragmentation was carried out on ions with charges of 2+ to 6+ and the top 20 parent ions with peak intensities greater than 20,000 were selected. Ion fragmentation was achieved using high-energy collisional dissociation (HCD) in combination with Orbitrap detection. Setting the dynamic exclusion time to 30 s, the automatic gain control was adjusted to 1E5 for MS1 and 2E4 for MS2.

#### 2.5.4. Cluster Analysis and Pathway Enrichment Analysis

The raw LC-MS/MS files were searched against the UniProt Protein Database (version 2.4, Thermo Scientific, San Jose, CA, USA) with the following parameters: precursor ion mass tolerance of 20 ppm, fragment ion mass tolerance of 20 ppm, and a maximum of two missed cleavages. The false discovery rate (FDR) was set at 1%. Differentially expressed proteins (DEPs) were identified based on a fold change (FC) greater than 1.3 between Pb-treated group and control group, with a significance threshold of *p*-value < 0.05. Functional annotation of DEPs was performed using the Gene Ontology (GO) and Kyoto Encyclopedia of Genes and Genomes (KEGG) databases. Within the GO framework, DEPs were categorized into three subcategories: biological process (BP), cellular component (CC), and molecular function (MF). Functional insights into the DEPs were further explored with GO and KEGG enrichment analysis. A heatmap was generated for data analysis and visualization using an online platform (https://www.bioinformatics.com.cn, accessed on 10 December 2024]). Protein–protein interaction (PPI) network analysis was conducted using the STRING database (https://string-db.org/ (accessed on 9 August 2025)). Markov Clustering (MCL) with the inflation parameter ˃ 3 was applied to the PPI network to identify functional modules. The PPI network and clusters were visualized using Cytoscape software (version 3.7.2).

### 2.6. Statistical Analysis

Data are presented as means ± SEM for all experimental groups. Comparisons among groups were analyzed using one-way or two-way ANOVA, followed by Tukey’s post hoc multiple-comparison test. Post hoc tests were performed only when the omnibus ANOVA yielded a statistically significant F-value (*p* < 0.05) and homogeneity of variance was confirmed. Comparisons between two groups were conducted using unpaired Student’s *t*-tests assuming equal variance.

## 3. Results

### 3.1. Early Postnatal Pb Exposure Induced Significant Autism-like Behaviors in Offspring

**Figure 1:**
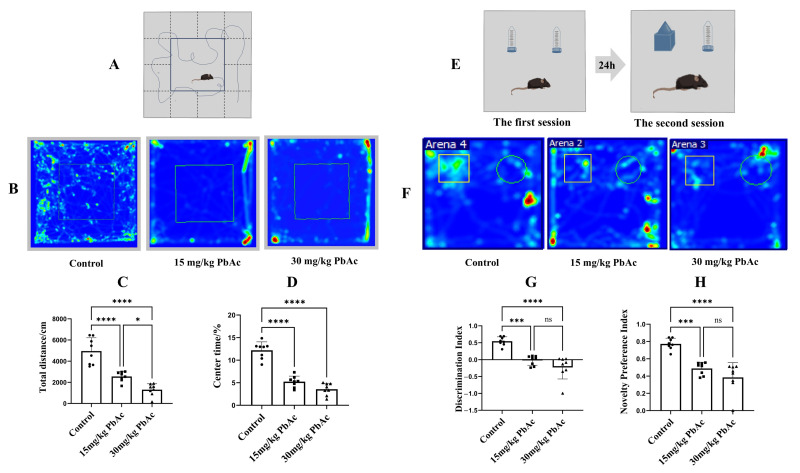
Effect of early postnatal Pb exposure on anxiety-like behaviors and recognition memory in mice offspring. (**A**) The trajectories in the open field test (OFT). (**B**) Heat maps of OFT. Circle represents Familiar Object, and square represents Novel Object. (**C**) The total distance traveled showed a significant decrease in the Pb-exposed groups. (**D**) A decrease in center time followed by Pb exposure. (**E**) Schematic diagram of the novel object recognition (NOR) test. (**F**) Heat maps of NOR test. Warmer colors (red) represent longer time spent and blue represents minimal time spent during the second session. (**G**) Pb-exposure offspring show a lower discrimination index, indicating the weaker recognition memory. (**H**) A decrease in novelty preference index in the Pb-exposed groups suggested the impaired short-term memory. * *p* ≤ 0.05, *** *p* ≤ 0.001, **** *p* ≤ 0.0001, “ns” represents not significance.

The OFT was conducted to assess psychomotor performance and anxiety-like behaviors (Figure 1A,B). The total distance traveled serves as an indicator of exploratory activity, while the time spent in the central zone measured anxiety, with lower values in both corresponding to increased anxiety-like behavior. Compared with control group, offspring exposed to Pb (15 mg/kg and 30 mg/kg) exhibited a markedly decrease in both the total distance traveled and the time spent in the central zone (Figure 1C,D), suggesting the presence of pronounced anxiety-like behaviors. The NOR test was employed to assess social recognition deficits in male offspring following postnatal Pb exposure. During the familiarization session, offspring were presented with two identical objects. In the subsequent test session, control offspring exhibited typical recognition performance, spending significantly more time to explore the novel object (Figure 1E,F). Conversely, offspring exposed to Pb (15 mg/kg and 30 mg/kg) showed impaired novel object recognition, spending more time exploring the familiar object than the novel one. This indicates significant cognitive impairment. Compared to controls, both the discrimination indices and novelty preference indices were significantly decreased in the Pb-exposed groups (Figure 1G,H).

**Figure 2:**
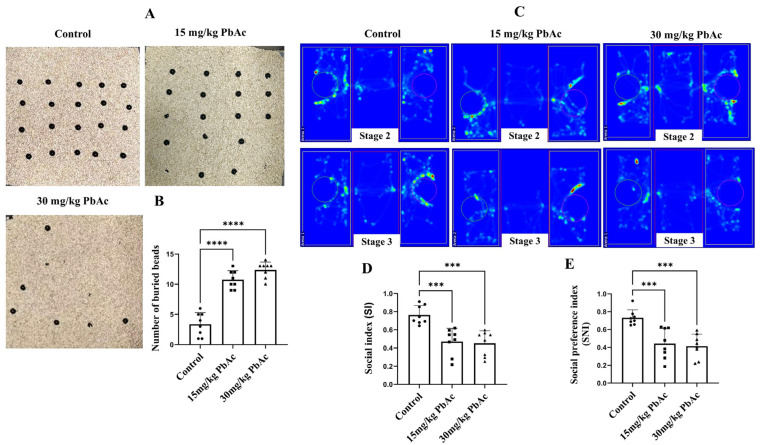
Effect of early postnatal Pb exposure on ASD-like repetitive behaviors and social behaviors in mice offspring. (**A**) Schematic of the marble buried test. (**B**) Pb-exposed mice buried a significantly greater number of marbles compared to the control mice. (**C**) Heat maps of male offspring in the three-chamber test. Maps illustrating the track of experimental animals in the test. In stage 2, the red circle is an empty cage, while the green circle contains a mouse; in stage 3, the red circle contains a stranger mouse, and the green circle contains a familiar mouse. Quantification of the social index (**D**) and the social novelty preference index (**E**) in the three-chamber test showed social deficits of Pb-exposed offspring. *** *p* ≤ 0.001, **** *p* ≤ 0.0001.

The marble burying test was employed to quantify restricted repetitive behaviors (Figure 2A). Compared to the control group, Pb-exposed offspring showed a significant increase in the number of marbles buried (Figure 2B, *p* < 0.0001). The three-chamber social test was performed to evaluate the social deficits in male offspring following postnatal Pb exposure. In the sociability stage, control mice spent more time in the chamber containing the stranger than in the empty chamber (Figure 2C). Accordingly, mice in the Pb exposure groups demonstrated a significant decrease in the SI and the SNI compared with those in the control group (*p* < 0.001, Figure 2C,D). These findings implied that mice exposed to Pb during the postnatal period display social deficits and repetitive behaviors, which are characteristic features of an ASD-like phenotype.

### 3.2. Early Postnatal Pb Exposure Induced Cerebellar Impairment

**Figure 3:**
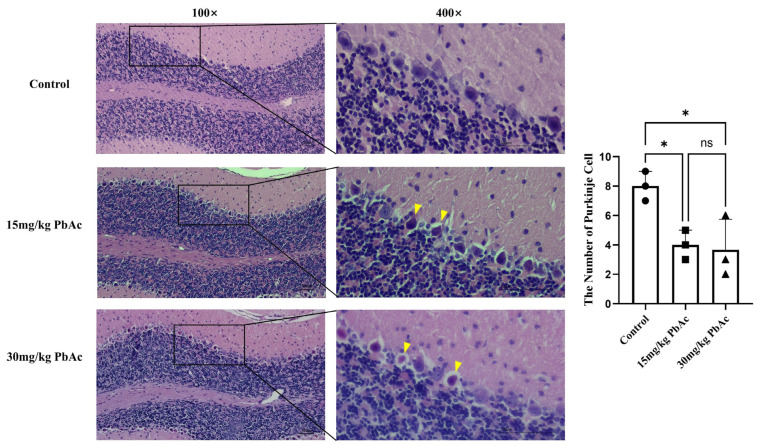
Altered structures and reduced numbers of Purkinje cells in the cerebellum. Purkinje cells were arranged neatly and tightly in control group, while deeply stained, pyknotic spindle cells were detected in both the 15 mg/kg PbAc and 30 mg/kg PbAc groups. Low-magnification images (×100); high-magnification images (×400). Yellow triangle represents pyknotic spindle cells. * *p* ≤ 0.05, “ns” represents not significance.

To investigate the effect of postnatal Pb exposure on the cerebellar morphology, H&E staining was performed. Histological analysis revealed that the cerebellar Purkinje cells in the control group were densely packed and arranged in an orderly manner, with distinct nucleoli. In contrast, the Pb-exposed groups exhibited a reduction in Purkinje cell number, disorganized cellular arrangement, and the presence of deeply stained pyknotic spindle-shaped cells (Figure 3). These results indicate that postnatal Pb exposure induced morphological alterations in the cerebellum of offspring mice.

### 3.3. Pb Levels in Cerebellum

**Figure 4:**
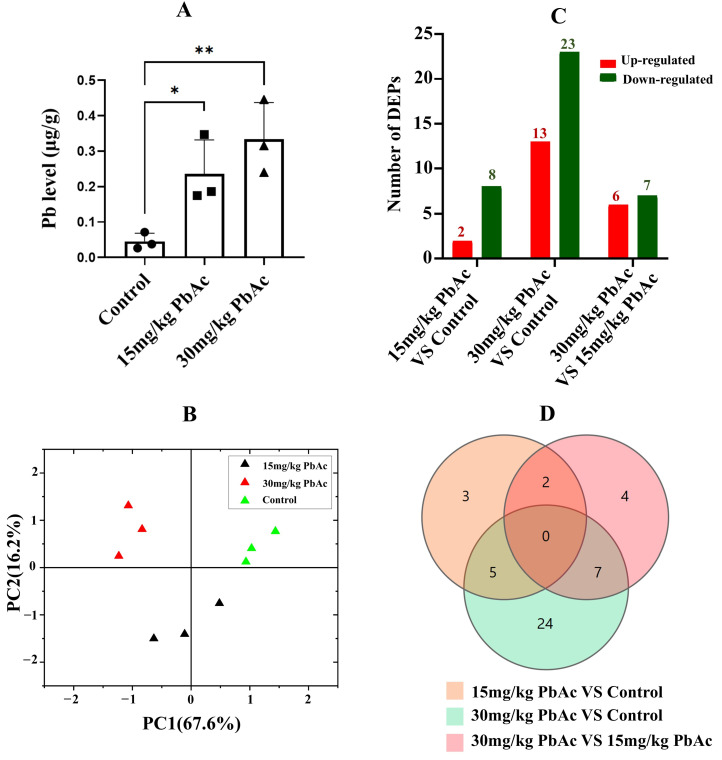
Pb levels in the cerebellum and Identification of cerebellar differentially expressed proteins (DEPs) following Pb exposure. (**A**) Pb levels in the cerebellum. (**B**) Principal component analysis (PCA) of the control and Pb-exposed groups. (**C**) Number of DEPs in each group. (**D**) Venn diagram analysis of overlapping and unique DEPs. * *p* ≤ 0.05, ** *p* ≤ 0.01.

After lead exposure from PND7 to PND21, Pb levels exhibited a significantly increase in the cerebellum in both the 15 mg/kg PbAc and 30 mg/kg PbAc groups compared to the control group (Figure 4A). Furthermore, cerebellar Pb levels demonstrated a clear dose–response relationship.

### 3.4. Protein Identification and Quantitative Analysis

**Figure 5:**
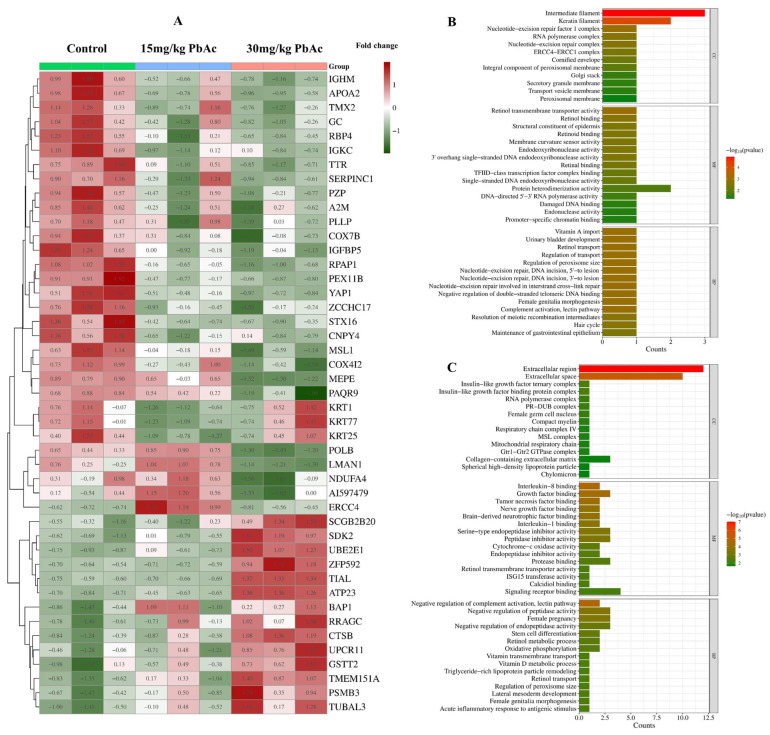
Heatmap and GO enrichment analyses of cerebellar DEPs. (**A**) Heatmap and cluster analysis of DEPs across the three groups. (**B**) Go enrichment analysis of DEPs (15 mg/kg PbAc vs. control group). (**C**) Go enrichment analysis of DEPs (30 mg/kg PbAc vs. control group).

Quantitative TMT-based proteomic analysis was applied for a proteome comparison between control and Pb-exposed groups. The MS system stability was supervised by quality control (QC) samples during the whole data-collecting period. A total of 7633 proteins were characterized by referencing the Mus Musculus database. Principal component analysis (PCA) revealed distinct separation between the control and Pb-exposed groups, with samples within each group exhibiting tight clustering based on principal component 1 (PC1) and 2 (PC2) (Figure 4B). Postnatal Pb exposure induced differential protein expression in the cerebellum, with a total of 41 proteins identified as DEPs compared to the control group. Subsequent cluster analysis was performed following the methodology described by Tang et al. [42] (Table S1, Figure 5A). Specifically, comparison of the 15 mg/kg PbAc group versus the control group identified 10 DEPs (2 upregulated, 8 downregulated). In contrast, the 30 mg/kg PbAc group versus the control group detected 36 DEPs (13 upregulated and 23 downregulated). Additionally, comparison between the 30 mg/kg PbAc group and 15 mg/kg PbAc group revealed 13 DEPs (6 upregulated and 7 downregulated, Figure 4C). Notably, Venn diagram analysis of the DEPs identified in these three group comparisons showed that no protein was commonly differentially expressed across all three pairwise comparisons (Figure 4D).

### 3.5. Go Enrichment Analysis and KEGG Pathway Analysis

GO functional enrichment analysis was performed to characterize the identified DEPs. Among the 10 DEPs in the 15 mg/kg PbAc group versus the control group, the top-ranked cellular component (CC) terms were primarily associated with intermediate filament and keratin filament. In the molecular function (MF), key terms involved in retinol transmembrane transporter activity and retinol binding, while biological process (BP) terms encompassed vitamin A import and urinary bladder development in the (Figure 5B). For the 36 DEPs in the 30 mg/kg PbAc group versus control, dominant CC terms localized to the extracellular region and extracellular space. Top MF terms centered on interleukin-8 binding and growth factor binding, whereas BP terms were enriched in negative regulation of complement activation and negative regulation of peptidase activity (Figure 5C).

**Figure 6:**
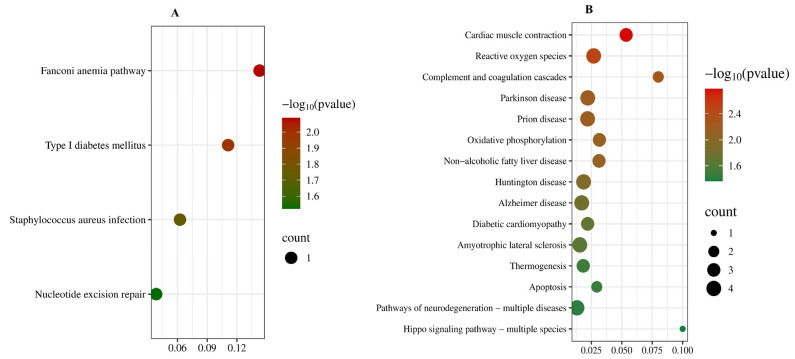
KEGG enrichment analysis of cerebellar DEPs following Pb exposure. (**A**) KEGG pathways of DEPs (15 mg/kg PbAc vs. control group). (**B**) KEGG pathways of DEPs (30 mg/kg PbAc vs. control group).

KEGG pathway enrichment analysis revealed significant biochemical pathways associated with DEPs, identifying 4 and 15 enriched pathways in the 15 mg/kg PbAc group and the 30 mg/kg PbAc group, respectively (Figure 6). Specifically, the 15 mg/kg PbAc group showed enrichment in Fanconi anemia pathway, type Ι diabetes mellitus, staphylococcus aureus infection, and nucleotide excision repair. The 30 mg/kg PbAc group exhibited five top pathways, including cardiac muscle contraction, reactive oxygen species, complement and coagulation cascades, Parkinson disease, and prion disease. These results implicate multiple pathways in Pb-induced autism-like social deficits and cognitive impairment.

### 3.6. PPI Network Construction and Hub Protein Identification

**Figure 7:**
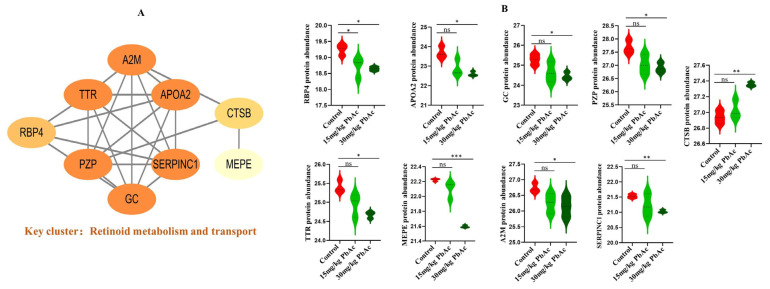
Protein–protein interaction (PPI) network analysis of target proteins. (**A**) Cluster Visualization (Key cluster: retinoid metabolism and transport). (**B**) Protein abundance of nine target proteins in retinoid metabolism and transport. * *p* < 0.05, ** *p* < 0.01, *** *p* < 0.001; ns, not significant.

To investigate functional connection among the identified pathways, all 41 DEPs were analyzed using STRING database to construct a PPI network. The resulting network focused on a key functionally distinct sub-network, that is retinoid metabolism and transport (Figure 7A). Alteration were observed in the retinoid metabolism and transport sub-network, which included nine DEPs: alpha-2-macroglobulin-P (A2M), transthyretin (TTR), apolipoprotein A-II (APOA2), pregnancy zone protein (PZP), vitamin D-binding protein (GC), antithrombin-III(SERPINC1), retinol-binding protein 4 (RBP4), cathepsin B (CTSB), and matrix extracellular phosphoglycoprotein (MEPE). Compared with the control group, the 30 mg/kg PbAc group exhibited a significant up-regulation in CTSB protein expression, while the expression of A2M, APOA2, PZP, MEPE, SERPINC1, and GC showed significant decreases (Figure 7B). These findings demonstrate functional enrichment of DEPs in critical signaling pathways, with retinoid metabolism and transport representing a major regulatory hub in the cerebellum.

## 4. Discussion

The cerebellum is among the most frequently implicated brain regions in ASD [43]. Evidence from cerebellar abnormalities in autistic patients, particularly Purkinje cells, has been strongly linked to ASD and is believed to underlie its symptomatic manifestations [22,23,44,45,46,47]. In parallel, accumulating evidence indicates that childhood Pb exposure induces neurological and behavioral abnormalities, significantly increasing the risk of neuropsychiatric disorders including ASD [2,3,16]. Consistent with these findings, both our previous and current studies demonstrate that Pb exposure significantly induced core ASD phenotypes in offspring, such as social deficits, repetitive behaviors, and impaired social cognition [47], along with significant impairments of Purkinje cells in cerebellum. As Pb can cross biological barriers and accumulate in the brain, we detected significantly elevated Pb levels in the cerebellum of Pb-exposed mice compared to controls, supporting a clear dose–response relationship. These findings further strengthen the correlation between Pb exposure and ASD-like traits. Nevertheless, the pathological mechanisms underlying Pb-induced ASD remain poorly understood. Given the established role of cerebellar impairment in ASD and its high susceptibility to environmental toxicants, we conducted a comprehensive cerebellar proteomic analysis in Pb-exposed offspring to identify associated molecular pathways.

A total of 45 DEPs were successfully identified, and multiple signaling pathways were enriched based on GO and KEGG analyses. Our PPI analysis highlighted the retinoid metabolism and transport pathway (Figure 7A) as being significantly enriched, underscoring its central role. This pathway involves key ASD-associated proteins such as RBP4, TTR, GC, and CTSB, which are associated with retinoid signaling. These results highlight the particular vulnerability of retinoid signaling to Pb exposure and suggest a potential mechanistic link to Pb-induced developmental impairment.

Retinol-binding protein 4 (RBP4), a newly identified adipokine, serves as the primary transporter of retinol and is related to the dysregulation of energy metabolism [48]. Furthermore, it functions as an inflammatory neurotrophic factor and represents a promising mediator within the fat-brain axis [49]. Recent studies indicate that RBP4 is permeable to the blood–brain barrier (BBB) and its expression increases concomitantly with BBB dysfunction. Previous clinical studies have demonstrated that a decreased RBP4 concentration in serum is associated with the autistic regression phenomenon and the severity of ASD [50]. Consistent with these observations, our proteomic analysis revealed a marked downregulation of RBP4 expression following exposure to both 15 mg/kg PbAc and 30 mg/kg PbAc (Figure 7, Table S1). These results suggest, for the first time, that Pb exposure may disrupt retinoid signaling and neuroimmune metabolic crosstalk via the suppression of RBP4, which may contribute to its neurodevelopmental toxicity. The dose-dependent reduction in RBP4 underscores its role as a sensitive biomarker of Pb-induced metabolic and neurological alterations. The retinol-RBP4 complex is secreted from the hepatocyte into circulation, facilitating retinol delivery to peripheral tissues, where it can be oxidized to retinoic acid (RA). In plasma, retinol-RBP4 circulates bound to transthyretin (TTR), which stabilizes the complex, reduces renal clearance, and enables RBP4 recycling after cellular retinol uptake [51]. Notably, our proteomic data also revealed a significant decrease in TTR expression in the cerebellum following Pb exposure. Given the well-documented association between disrupted RA signaling and ASD [30,31], as well as evidence that RA supplementation improves social deficits and neural connectivity in ASD models [33,52], the concurrent downregulation of RBP4 and TTR strongly implies that Pb neurotoxicity impairs retinoid transport and signaling. Together, these findings reveal a novel mechanism through which Pb exposure disrupts RA-mediated neurodevelopmental processes in the cerebellum via the coordinated suppression of RBP4 and TTR, providing new mechanistic insights into the contribution of Pb exposure to the etiology of ASD.

Maternal vitamin D deficiency during pregnancy has been identified as a potential risk factor for ASD in offspring [53]. Supporting this association, lower serum vitamin D levels have been consistently observed in children with ASD, suggesting that vitamin D insufficiency may serve as an environmental contributor to the development of ASD [54,55,56]. In our cerebellar proteomic analysis, we found a significant decrease in vitamin D-binding protein (GC) following exposure to 30 mg/kg PbAc. GC plays a crucial role in vitamin D transport and storage and helps regulate serum vitamin D levels [57]. Notably, recent evidence indicates that a specific GC isoform (GC1f) may serve as a biomarker for ASD severity [58]. These findings suggest a potential mechanism through which Pb exposure could contribute to cerebellar abnormalities associated with ASD.

Of the DEPs identified within the retinoid metabolism and transport pathway, cathepsin B (CTSB) was the only one significantly upregulated after Pb exposure. CTSB is a multifunctional cysteine protease uniquely expressed in various cell types, including microglia in the developing brain, where it plays a critical role in regulating phagocytic activity during synaptic pruning. Abnormal expression of CTSB may impair microglia function, particularly at early stages of neurodevelopment when programmed pruning is most active [59]. Importantly, elevated CTSB levels have been recently associated with increased risk of ASD [60]. Thus, the specific upregulation of CTSB following Pb exposure suggests a potential mechanistic link between environmental Pb exposure and disrupted neurodevelopmental processes, possibly mediated through impaired retinoid signaling and microglial dysfunction.

It is noted that GO analysis revealed that inconsistent pathways are enriched by different doses of Pb exposure. Low-dose Pb exposure suggests early disruption of cell structure and developmental signals, such as by compromising the integrity of the cytoskeleton (e.g., intermediate filaments) and early interference with neuronal structure and function [61]. On the other hand, high-dose Pb exposure is associated with intense neuroinflammation and immune dysregulation. Pb is a known pro-inflammatory agent, and the release of pro-inflammatory cytokines can impair neuronal function [62]. Enrichment of the “negative regulation of complement activation” pathway suggests a malfunction in the immune system’s self-regulatory mechanisms. This dysregulated inflammatory response may cause sustained damage to the developing brain, affecting synaptic pruning and neural circuit formation, leading to behavioral phenotypes similar to ASD.

While our study offers valuable insights into ASD etiology with Pb exposure and has methodological strengths, we acknowledge several limitations. Due to the substantially higher prevalence of ASD in males than in females (approximately 4:1), the current study was confined to male offspring. It has been reported that female offspring in ASD models demonstrate more pronounced social deficits, repetitive behaviors, and anxiety-like behaviors [63,64]. Thus, further investigation of ASD-related traits in female subjects and examination of potential sex differences are warranted. Additionally, although behavioral tests such as the three-chamber social test and open field test have been widely used in the evaluation of autistic-like behaviors, they still cannot be regarded as the gold standard for ASD-like behavior assessment. Observations in such behavioral studies are often susceptible to environmental variability during testing. The three-chamber social test, as a widely adopted outcome-focused paradigm, demonstrates that observed social impairments may be partly attributable to confounding variables such as cognitive deficits, compromised recognition memory, or anxiety-related behavioral components. Thus, future studies should implement more refined social testing paradigms or incorporate neurobiological markers to accurately identify the core mechanisms that underlie social deficits.

## 5. Conclusions

In summary, based on the mouse model of early postnatal Pb exposure, this study investigated the ASD-like behaviors in offspring following, histological changes in the cerebellum, and associated proteomic alterations. These results suggest a particular susceptibility of the retinoid signaling pathway to early postnatal Pb exposure, implying a possible association Pb-induced ASD-like symptoms. This disruption may occur through the coordinated dysregulation of RBP4, TTR, GC, and CTSB, providing new perspectives on the role of environmental factors in the pathogenesis of ASD and exploring potential therapeutic targets.

## Data Availability

All relevant data of this study are given in the manuscript. Additional data will be provided upon request.

## Supplementary Materials

The following supporting information can be downloaded at: https://www.mdpi.com/article/10.3390/cimb47100861/s1.
