# Supplementary material for: Contributions of Retinoid Signaling to Autism-like Behaviors Induced by Early Postnatal Lead Exposure in the Mouse Cerebellum
Source: Curr Issues Mol Biol. 2025 Oct 18;47(10):861. doi: 10.3390/cimb47100861 (PMC12563089; doi:10.3390/cimb47100861)
Supplement: Supplementary file 1 [file cimb-47-00861-s001.zip › cimb-3911623-supplementary.pdf]

Table S1. List of cerebellar DEPs identified after postnatal Pb exposure.

No.	Accessions	Protein name	Gene name	15mg/kg PbAc group *VS* control group		30 mg/kg PbAc group *VS* control group	
				FC	*Q* value	FC	*Q* value
1	Q8BQ47	Protein canopy homolog 4	CNPY4	-2.58▼	0.039	-2.35▼	0.042
2	P01837	Immunoglobulin kappa constant	IGKC	-2.35▼	0.033	-2.21▼	0.041
3	Q80TE0	RNA polymerase II-associated protein 1	RPAP1	-1.41▼	0.007	-1.64▼	0.004
4	Q00724	Retinol-binding protein 4	RBP4	-1.44▼	0.049	-1.52▼	0.032
5	Q9Z210	Peroxisomal membrane protein 11B	PEX11B	-1.34▼	0.036	-1.41▼	0.036
6	P09813	Apolipoprotein A-II	APOA2	-1.72●	0.067	-2.17▼	0.049
7	P01872	Immunoglobulin heavy constant mu	IGH-6	-1.57●	0.074	-1.97▼	0.050
8	P21614	Vitamin D-binding protein	GC	-1.51●	0.129	-1.80▼	0.042
9	Q61838	Pregnancy zone protein	PZP	-1.56●	0.072	-1.73▼	0.044
10	P56393	Cytochrome c oxidase subunit 7B, mitochondrial	COX7B	-1.40●	0.106	-1.71▼	0.044
11	P46938	Transcriptional coactivator YAP1	YAP1	-1.50●	0.054	-1.69▼	0.040
12	P07309	Transthyretin	TTR	-1.36●	0.076	-1.66▼	0.021
13	Q6PDM1	Male-specific lethal 1 homolog	MSL1	-1.29●	0.051	-1.63▼	0.008

14	Q8BVI5	Syntaxin-16	STX16	-1.54●	0.051	-1.56▼	0.044
15	Q8K4L6	Matrix extracellular phosphoglycoprotein	MEPE	-1.09●	0.180	-1.54▼	0.000
16	Q9D710	Thioredoxin-related transmembrane protein 2	TMX2	-1.27●	0.254	-1.50▼	0.021
17	Q9D0F3	Protein ERGIC-53	LMAN1	1.20●	0.109	-1.49▼	0.051
18	Q6GQT1	Alpha-2-macroglobulin-P	A2M	-1.37●	0.078	-1.48▼	0.047
19	P32261	Antithrombin-III	SERPINC1	-1.21●	0.307	-1.42▼	0.002
20	Q9DCU2	Plasmolipin	PLLP	-1.17●	0.367	-1.35▼	0.033
21	Q6TCG2	Membrane progesterone receptor epsilon	PAQR9	-1.06●	0.026	-1.34▼	0.034
22	Q91W29	Cytochrome c oxidase subunit 4 isoform 2, mitochondrial	COX4I2	-1.12●	0.194	-1.32▼	0.011
23	Q9ESX4	Zinc finger CCHC domain-containing protein 17	ZCCHC17	-1.29●	0.009	-1.31▼	0.021
24	Q07079	Insulin-like growth factor-binding protein 5	IGFBP5	-1.24●	0.018	-1.31▼	0.013
25	Q8K409	DNA polymerase beta	POLB	1.05●	0.043	-1.28▼	0.001
26	Q62425	Cytochrome c oxidase subunit NDUFA4	NDUFA4	1.05●	0.460	-1.24▼	0.077
27	Q6IFZ6	Keratin, type II cytoskeletal 1b	KRT77	-1.40▼	0.041	-1.03●	0.830
28	P04104	Keratin, type II cytoskeletal 1	KRT1	-1.34▼	0.041	-1.03●	0.831

29	Q8VCW2	Keratin, type I cytoskeletal 25	KRT25	-1.34▼	0.043	-1.08●	0.469
30	Q922M7	Ashwin	AI597479	1.19▲	0.050	-1.19●	0.176
31	Q9CWQ3	Mitochondrial inner membrane protease ATP23 homolog	ATP23	1.09●	0.108	2.82▲	0.000
32	Q8BHZ4	Zinc Finger Protein 592	ZFP592	-1.02●	0.453	1.98▲	0.030
33	P70318	Nucleolysin TIAR	TIAL1	-1.01●	0.548	1.89▲	0.000
34	P52482	Ubiquitin-conjugating enzyme E2 E1	UBE2E1	1.11●	0.247	1.61▲	0.003
35	Q3UX10	Tubulin alpha chain-like 3	TUBAL3	1.24●	0.090	1.60▲	0.045
36	Q6V4S5	Protein sidekick-2	SDK2	1.08●	0.285	1.55▲	0.004
37	Q6GQT5	Transmembrane protein 151A	TMEM151A	1.18●	0.218	1.53▲	0.001
38	Q9JI02	Secretoglobin family 2B member 20	SCGB2B20	1.05●	0.653	1.49▲	0.026
39	Q9CPX8	Cytochrome b-c1 complex subunit 10	UQCR11	1.02●	0.838	1.33▲	0.020
40	P10605	Cathepsin B	CTSB	1.06●	0.372	1.33▲	0.004
41	Q61133	Glutathione S-transferase theta-2	GSTT2	1.11●	0.350	1.33▲	0.041
42	Q99K70	Ras-related GTP-binding protein C	RRAGC	1.16●	0.204	1.32▲	0.050
43	Q99PU7	Ubiquitin carboxyl-terminal hydrolase BAP1	BAP1	1.28●	0.213	1.30▲	0.031

44	Q9QZD4	DNA repair endonuclease XPF	ERCC4	1.65▲	0.019	1.02●	0.502
45	P97411	Islet cell autoantigen 1	ICA1	1.30▲	0.047	-1.20●	0.180

▲Up-regulated, ▼Down-regulated, ●Not-significant
